# The Iowa Medical Journal

**Published:** 1855-09

**Authors:** 


THE IOWA MEDICAL JOURNAL.

The present number closes the second volume of this work. To be able to say that our labors have been so generally approved is a circumstance truly gratifying indeed. We derive this assurance from various sources and in different ways—by letters from many of our kind patrons, whose good opinion we regard as valuable,—from the press,—and from our State organization. This certainly is encouraging and very flattering to our efforts. We do assure our readers that the task is no light one, because performed in the midst of pressing professional duties which every medical practioner knows is inexorable. We made promises at first and repeatedly renewed them that our best efforts, under the circumstances, should be rendered to make it useful. How far we have kept the faith we only know from the testimony above referred to.

Without remuneration we have labored at a task of no ordinary magnitude and we are still disposed to continue the effort with increased zeal. But we cannot labor unless the vitalizing element be supplied. And yet we ask for nothing but the material substance to sustain the Journal itself. We believe that our patrons who are generally zealous and intelligent as elsewhere found, fully appreciate our position and will at once supply all arrears and add new members to our respectable list of subscribers.

The medical man who takes a deep interest in his profession, and aims to keep pace with medical progress, would not for any consideration, dispense with the monthly, bi-monthly, or quarterly visits of a journal. If he did not take a medical periodical he would not consider himself, nor would others consider him, as emulous of distinction and the capacity for usefulness, as is demanded by the wants of society. Not only should we patronise these periodicals because of the manifest benefit derivable from their pages, but in a particular manner should the profession of a State sustain its *own* Journal. Every medical man in this State should consider himself a share-owner in the work and that it belongs to him in common with the profession. It is his organ when he finds occasion to express his views upon any medical topic, at the same time that he gleans therefrom the views and opinions of his brethren. Who can estimate the benefits of a good journal in the midst of a profession scattered over the country, and especially one filling up and improving as rapidly as ours? It establishes a bond between the members of the medical body and it unites them in a common interest. It diffuses its influences equally among all and gives to the profession a character by embodying its experience and expressing its wishes. When we speak of the profession of any one State, we presume it is to a certain extent organized as such and when the profession of one district of country desires to speak to another, it does so through its organ which is reflected from the pages of the periodical in their midst. The progress of medical literature, is from time to time made known, and every new improvement immediately announced to them. It awakens a desire on the part of medical men to contribute to our medical literature, because the report of a case, with its treatment, finds a parallel perhaps in the experience of another. This will superinduce a closer observation and a more careful record of their cases.

These are a few of the many advantages arising from the periodicals of the country, which are well understood and appreciated by the mass of the profession.

We will estimate the appreciation, by our subscribers, of our former efforts by the promptness with which they will order their subscription renewed, those which expire with the present volume, which will be most gratifying, but more particularly so provided a new subscriber accompanies.

The next number will appear about the first of November, and then once every two months during the coming year, as the last volume. We shall make an effort to make the next volume an improvement upon the last, and our best efforts will be exerted to promote the interest and uphold the dignity of the profession.

